# Oral status and dental treatment needs in patients with Epidermolysis Bullosa - a cross-sectional study

**DOI:** 10.1007/s00784-026-06779-x

**Published:** 2026-04-10

**Authors:** Sebastián Véliz, Pedro Diz-Dios, Colomba Besa-Witto, Susanne Krämer

**Affiliations:** 1 Department of Periodontology, Dental Clinic, Sigmund Freud University, Vienna, Austria; 2 Facultad de Medicina y Odontoloxia, Universidad de Santiago de Compostela, Santiago de Compostela, Spain; 3 Department of Dermatology, Medical faculty and medical centre, University of Freiburg, Freiburg im Breisgau, Germany; 4 Special Care Dentistry Unit, Facultad de Odontología, Universidad de Chile, Olivos 943, Independencia, Santiago, Chile

**Keywords:** Epidermolysis Bullosa, Oral health, Dental treatment needs

## Abstract

**Objective:**

To determine the oral health status and dental treatment needs in a cohort of children and adults with different types of Epidermolysis Bullosa (EB).

**Materials and methods:**

A cross-sectional study was designed. 101 participants with EB (EB Simplex *n* = 26, Junctional EB = 6, Dominant Dystrophic EB (DDEB) = 20, Recessive Dystrophic EB (RDEB) = 47, Kindler EB = 2) were assessed including simplified debris index (DI-S), Basic Periodontal Examination (BPE), Decay/Missing/Filled Teeth index (DMFT/deft index) and Orthodontic index of complexity, outcome and need (ICON). Treatment needs were classified into preventive, periodontics, restorative, endodontics, orthodontics, prosthodontics, oral surgery, oral pathology, oral radiology, speech therapy and others. Descriptive and statistical analyses were performed.

**Results:**

The sample showed a Debris Index of 2.04 ± 0.8, with higher levels in participants with recessive dystrophic EB (2.34 ± 0.7). 15.1% of the total sample (*n* = 15) showed a BPE value of 3 or 4. DMFT was 11.2 ± 0.1, while 30.1% (*n* = 28/93) showed an ICON value of 43 or higher. Treatment needs were high, with a median of 3 referrals per patient, with the highest needs in restorative dentistry (*n*=55, 54.5%), prosthodontics (*n*=52, 51.5%), and speech/oral function therapy (*n*=43, 42.6%). By EB type, junctional EB (3.67±0.52) and recessive dystrophic EB (3.34±1.03) showed the highest need for referrals.

**Conclusion:**

Oral health status among people living with EB differed across major types. Patients with recessive dystrophic EB were associated with poorer oral hygiene, a higher caries experience and more missing teeth, whereas those with junctional EB were associated with a higher number of restored teeth. On the opposite, those with simplex EB and dominant dystrophic EB were associated with more favourable oral health indicator. The greatest referral needs were for restorative dentistry, prosthodontics and speech therapy, with higher referral needs observed among EB subtypes with high risk of oral disease (recessive dystrophic EB, junctional EB and Kindler EB). Older age was associated with periodontics and prosthodontics referral needs, while male participants were associated with a higher need for speech therapy.

**Clinical relevance:**

This research seems to indicate that patients with recessive dystrophic EB and junctional EB are at high risk of oral disease, requiring referrals to at least 3 different dental specialities. In addition to paediatric dentists, special care dentists and orthodontists, dental teams caring for patients with EB should include specialists in restorative dentistry and prosthodontics, with access to consultants in speech and language therapy, endodontics and periodontics.

**Supplementary Information:**

The online version contains supplementary material available at 10.1007/s00784-026-06779-x.

## Introduction

Epidermolysis Bullosa (EB) is a genodermatosis characterised by skin and mucosal fragility, due to genetic variants that compromise structural proteins of the dermo-epidermal junction [1, 2]. At least 16 genes have been linked to EB, causing a wide range of clinical phenotypes. The present classification considers four major types: EB Simplex (EBS), Junctional EB (JEB), Dystrophic EB (DEB), and Kindler EB (KEB), with 35 subtypes, ranging from mild to severe [1, 2].

As a mechanobullous disorder, the primary clinical characteristic is the presence of skin and mucosal lesions secondary to shear forces. The blistering is caused at different cleavage levels according to the major EB type, with EBS presenting cleavage within the basal layer of the skin, JEB in the lamina lucida of the basement membrane zone, DEB in the dermis, and KEB affecting intracellular protein of focal adhesion (kindlin-1) [1, 2]. These lesions include, but are not limited to, ulcers, blisters, and wounds [1, 2]. Clinical phenotypes vary according to the type and subtype of the condition. People with EBS can present limited lesions and nail atrophy, while severe forms of EBS can be associated with muscular dystrophy. Junctional EB can include granulation tissue, alopecia and failure to thrive. RDEB can be associated with chronic pain, pseudosyndactyly, nail dystrophy, and acral deformities, high incidence of Squamous Cell Carcinoma (SCC), gastrointestinal and nutritional anomalies, ocular findings, anaemia, cardiomyopathy and renal compromise. KEB can present mixed levels of blistering, as well as poikiloderma and skin atrophy [1–5].

Oral findings in EB also vary according to the type and subtype [6]. People living with recessive dystrophic EB (RDEB) present an increased prevalence of oral lesions and strictures, such as microstomia, ankyloglossia and vestibule obliteration. In addition, atrophic skin and mucosa are observed, with loss of tongue papillae and palatal rugae. These oral characteristics, in association with a soft diet and pseudosyndactyly, lead to extensive plaque deposits and poor oral hygiene, showing a higher caries prevalence [6–9]. Patients with JEB can present perioral granulation tissue, Syndromic Amelogenesis Imperfecta (SAI), crown resorption and teeth retention [6, 10, 11], while people with Kindler EB can present early onset periodontal diseases, oral strictures and a pitted pattern of SAI [6, 12, 13].

Dental treatment in patients living with EB is complex, as skin and mucosal fragility, systemic and oral manifestations including blisters, ulcers, bleeding and pain, affect proper oral hygiene, nutrition and dental treatment. Multidisciplinary management throughout the life cycle is required and must be tailored to the type, subtype, and clinical features of each patient [14]. Consequently, different dental specialities have reported their experience in the management of specific conditions and complications: special care dentistry and paediatric dentistry manage clinical and behavioural challenges, orthodontics treats malocclusions related to scarring, periodontics treats periodontal diseases in KEB patients as well as gingival tissue; prosthodontics and implantology support oral rehabilitation in cases of tooth loss or associated amelogenesis imperfecta; oral surgery can be required for contractures management, while endodontics and oral radiology can require modified protocols due to limited mouth opening and mucosal fragility [6, 11, 15–25].

Tools such as the oral health care pathways for patients with EB from the European Reference Network for Rare Skin Diseases [14] and the Clinical Practice Guidelines: Oral health care for children and adults living with EB [6] have helped to guide professionals in providing adequate clinical management and treatment. Although multidisciplinary dental care is recommended, more information on specific specialist referrals is needed. To date, the dental treatments needs for children living with EB have been described in a scoping review of 45 case reports, published in 2024. This study concluded that dental management is complex and requires specialised care, focusing mainly on individualised early preventive care [26]. No similar studies were found in adults. Therefore, this study aims to determine the oral health status and dental treatment need (specialist referrals) in a cohort of children and adults with different types of EB.

## Materials and methods

### Study description

A cross-sectional study was conducted at the Faculty of Dentistry, University of Chile, in association with DebRA (Dystrophic Epidermolysis Bullosa Research Association) Chile, a nationwide charity caring for all patients with EB in the country and holding the national register. A convenience sample was established, including all patients meeting the inclusion criteria from January 2023 to December 2024. All patients registered at DebRA Chile were invited to participate during their regular dental check-ups. The possible number of participants was established at 192, according to the prevalence of EB previously reported [27].

### Ethical statements

This project was approved by the Ethics Committee of the North Metropolitan Health Service, Chile (002/2023), and did not receive any funding. This report was written following the Strengthening the Reporting of Observational Studies in Epidemiology guidelines for cohort studies (STROBE)[28]. All participants signed informed consent for their participation, and patients under 18 years old were asked to sign an assent form together with their guardians' consent. 

### Inclusion and exclusion criteria

Inclusion criteria: Participants with genetically confirmed EB diagnoses, (all EB types and subtypes) diagnosed by a dermatologist according to the criteria published by Has et al. (2020) [1], with no age or any other restriction who agreed to participate by signing an informed consent/assent. Exclusion criteria: lack of cooperation for oral examination, for example young patient not willing to open the mouth.

### Clinical and treatment needs assessment

The examination included routine extraoral and intraoral assessments, using the following parameters:

- Simplified debris index (DI-S): Developed by Green and Vermillion in 1964. The simplified version assesses the presence of soft debris covering indexes teeth for permanent (11, 16, 26, 31, 36, 46) and primary dentition (54, 61, 64, 75, 82, 85). Typically, the buccal surface of the upper teeth and the lingual surface of the lower teeth are assessed. Scores are 0 (No debris or stains can be observed), 1 (soft debris covers less than ⅓ of the surface, 2 (soft debris covers between ⅓ and ⅔ of the surface) and 3 (soft debris covers more than ⅔ of the surface). The score is the result of the sum of the debris score per tooth surface divided by the number of surfaces examined. The total index can be categorised as good (between 0.0 and 0.6), fair (between 0.7 and 1.8) and poor (between 1.9 and 3.0) [29].
- Basic Periodontal Examination (BPE) [30]: A World Health Organisation (WHO) BPE probe is used to assess periodontal status. The mouth is divided into sextants, and to qualify, the sextant must have at least two teeth. All the teeth present in mouth were assessed in six sites per tooth (3 vestibular/buccal sites: distobuccal, buccal and mesiobuccal; and 3 lingual/palatal sites: distolingual/palatal, lingual/palatal and mesiolingual/palatal). The probe should walk around in the periodontal pocket, and the highest score for each sextant should be recorded. Scores include 0 (< 3.5 mm pockets, no calculus or bleeding), 1 (< 3.5 mm pockets, no calculus, bleeding on probing), 2 (< 3.5 mm pockets, supra or subgingival calculus), 3 (probing depth between 3.5 and 5.5 mm) and 4 (probing depth > 5.5 mm). For those patients with codes 3 or 4, a complete periodontal charting was required using a UNC (University of North Carolina) periodontal probe, and periodontal staging (I to IV) and grading (A, B or C) was established according to the parameters of the current periodontal classification [31].
- Decayed/Missing/Filled Teeth Index (DMFT/deft): This index is the sum of the individuals’ decayed (D), missing (M) and filled (F) teeth in the permanent teeth. It ranges from 0 to 28 and includes all teeth except for the third molars. For primary dentition, the M for missing is changed to e, referencing “extracted”, all with lowercase letters (deft), ranging from 0 to 20 [32].
- Orthodontic index of complexity, outcome and need (ICON). This index considers five parameters: aesthetic, upper arch crowding/spacing, incisor open bite, incisor overbite, buccal segment anteroposterior. Each parameter is assessed from 0 to 5, and multiplied by a weighting factor. Final values in 43 points or higher are considered to need orthodontic treatment. Complexity of treatment is categorised as easy (< 29 points), mild (29–50), moderate (51–63), difficult (64–77) and very difficult (> 77 points) [33].

After the assessment was completed, dental treatment needs were established according to the criteria in the supplementary Table 1. The data was analysed by EB type. Dominant (DDEB) and recessive dystrophic EB (RDEB) were analysed independently. Additionally, the classification proposed by Krämer et al. (2024) according to the risk of oral disease was used to analyse the data. This classification considered “high risk” (RDEB severe/ intermediate/ inversa, JEB and KEB), “moderate risk” (DDEB, EBS severe, RDEB localised) and “low risk” (EBS localised and moderate) [14].

### Risk of bias

To reduce the risk of bias, each participant was comprehensively assessed by one of two calibrated examiners (SV, CB; inter-rater Cohen’s Kappa = 0.928, JASP 0.19.0.0). Calibration was performed prior to data collection through a team training session and later, a set of 12 pilot participants, including different EB subtypes (EBS, JEB, DDEB and RDEB) were independently evaluated, compared and resolved the discrepancies until full agreement on operational definitions. No KEB participants were included during the calibration process due to the low prevalence in the country. These participants were subsequently re-evaluated and included in the final study sample, and all analyses were conducted using the final assessment. Although each patient was examined by only one examiner, any uncertainty during the process was first discussed between the two calibrated examiners, and only in case of disagreement, and a third party (SK) was consulted to reach a final consensus.

### Statistical analysis

The results were presented using descriptive statistics, including percentages, standard deviation, median and range. Missing data was properly addressed in the corresponding section, handled using complete-case analysis. No imputation was performed. The KEB group was excluded from analytic statistics due to the low number of participants (*n* = 2). Data distribution was analysed using the Shapiro-Wilk test. Due to the non-normal distribution, analysis was performed using the Kruskal-Wallis test with Dunn’s post-hoc pairwise test, as well as Fisher’s exact test with Bonferroni post-hoc pairwise test. Adjusted p-values were obtained by multiplying each unadjusted p-values by the number of variables analysed, with values higher than 1 truncated to 1. For age (as a continuous variable) and gender analysis, a binary logistic regression with an estimated OR (95% CI) and p-values with Bonferroni correction was performed. Adjusted logistic regression analyses were considered; however, due to the limited sample size and small number of events in several categories, the team considered that its use would have been statistically unstable and at high risk of overfitting. Therefore, we chose to present crude analyses and results interpreted descriptively. Statistical significance was considered when the *p*-value was < 0.05 (two-sided). The RStudio 2025.05.0 software was used for data analysis.

## Result

A total of 101 participants were examined, corresponding to the 52.6% of the patients in the national DebRA registry (n = 192) (Suppl. Figure 1). Considering a confidence level of 95% and an expected proportion of 50%, this sample size allows proportions to be estimated with a margin of error of approximately 6.7%. Gender distribution considered 54 men (53.4%) and 47 women (46.5%), no patient declared themselves as diverse. Average age was 22.2 years (SD: 15.4, range: 2–74). EB subtype, age and gender are presented in Table 1.

**Table 1:** Participants' description: EB Type and subtype, age and gender

EB type and Gene affected	EBS			JEB	DEB		KEB
					DDEB	RDEB	
	*KRT5* (*n*=20)	*PLEC* (*n*=5)	*KRT14* (*n*=1)	*LAMB3* (*n*=6)	*COL7A1* (*n*=20)	*COL7A1* (*n*=47)	*FERMT1* (*n*=2)
Subtypes	Localised (*n*=12) Intermediate (*n*=8)	Intermediate with muscular dystrophy (*n*=5)	Intermediate (*n*=1)	Intermediate (*n*=3) Severe (*n*=3)	Localised (*n*=14) Intermediate (*n*=2) Pruriginosa (*n*=4)	Localised (*n*=1) Intermediate (*n*=17) Severe (*n*=27) Inversa (*n*=2)	*n*=2
Age (years) [mean ± SD (range)]	32 ±19.4 (4-65)	13.8 ±11.1 (6-33)	4	12 ±2.9 (8-16)	21.4 ±16.6 (6-74)	20.8 ±12.5 (2-57)	29.5 ± 13.4 20-39
Gender (male/female/diverse) [*n* (%)]	13 (65) 7 (35) 0 (0)	3 (60) 2 (40) 0 (0)	0 (0) 1 (100) 0 (0)	3 (50) 3 (50) 0 (0)	10 (50) 10 (50) 0 (0)	25 (53.1) 22 (44.7) 0 (0)	0 (0) 2 (100) 0 (0)

***Abbreviations:*** *EB* epidermolysis bullosa, *EBS* EB Simplex, *JEB* Junctional EB, *DDEB* Dominant Dystrophic EB, *RDEB* Recessive Dystrophic EB, *KEB* Kindler EB

### Debris index-simplified (DI-S)

**Fig. 1:**
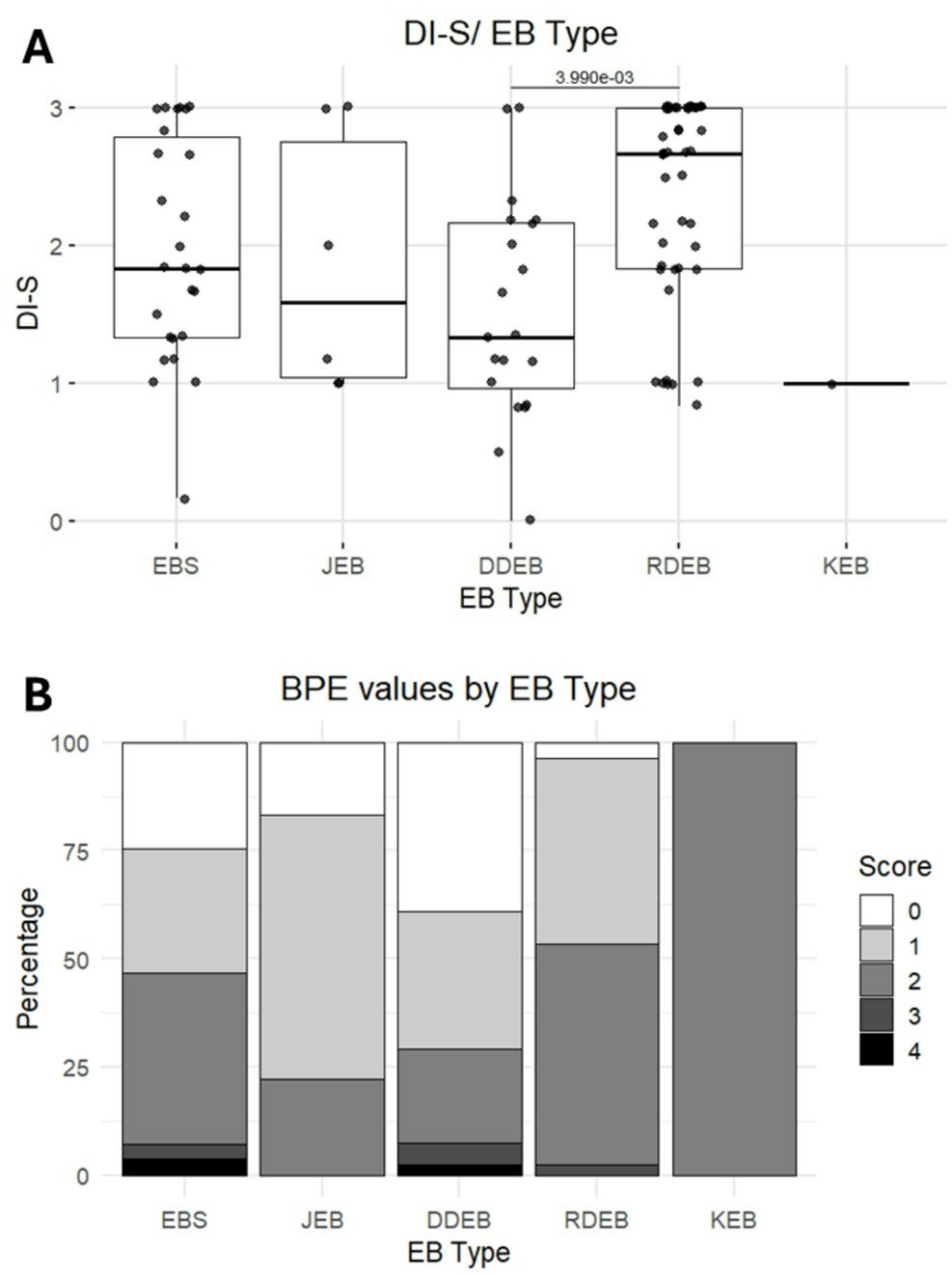
(**A**) Boxplot showing the distribution of the Debris Index Simplified (DI-S) by EB type. (**B**) Stacked bar chart with the basic periodontal examination values (BPE) by EB type. Decay, missing, filled teeth index

The average DI-S score for the complete cohort was 2.04 ± 0.8. The worst hygiene index was observed on patients with RDEB (2.34 ± 0.7), improving slightly in those with EBS (1.98 ± 0.8) and JEB (1.86 ± 0.9). Participants with DDEB showed better hygiene scores, with an average of 1.53 ± 0.8, while the lowest score was on the only patient with KEB assessed: 1. Statistical significance was found among EB types (p = 0.00739, Kruskal-Wallis/Dunn’s post-hoc), particularly when comparing DDEB-RDEB (p = 0.0039, Kruskal-Wallis/Dunn’s post-hoc) (Fig. 1A, Supplementary Table 2)

### Basic periodontal examination, periodontal stage and grade

According to BPE, 15 (15.1%) of 99 participants assessed had values of 3 or 4. These values were distributed among five patients with EBS, four with DDEB and six with RDEB. Those 15 participants were later thoroughly assessed, with one presenting gingival hyperplasia, four were considered stage 1, six stage 2, two stage 3 and two stage 4; while three were grade A, eight grade 2 and three grade 3. No statistical significance was found when the presence of periodontitis was analysed (*p* = 0.6746, Fisher’s Exact Test) (Fig. 1B, Supplementary Table 3).

### Decayed- missing- filled teeth index (DMFT)

**Fig. 2:**
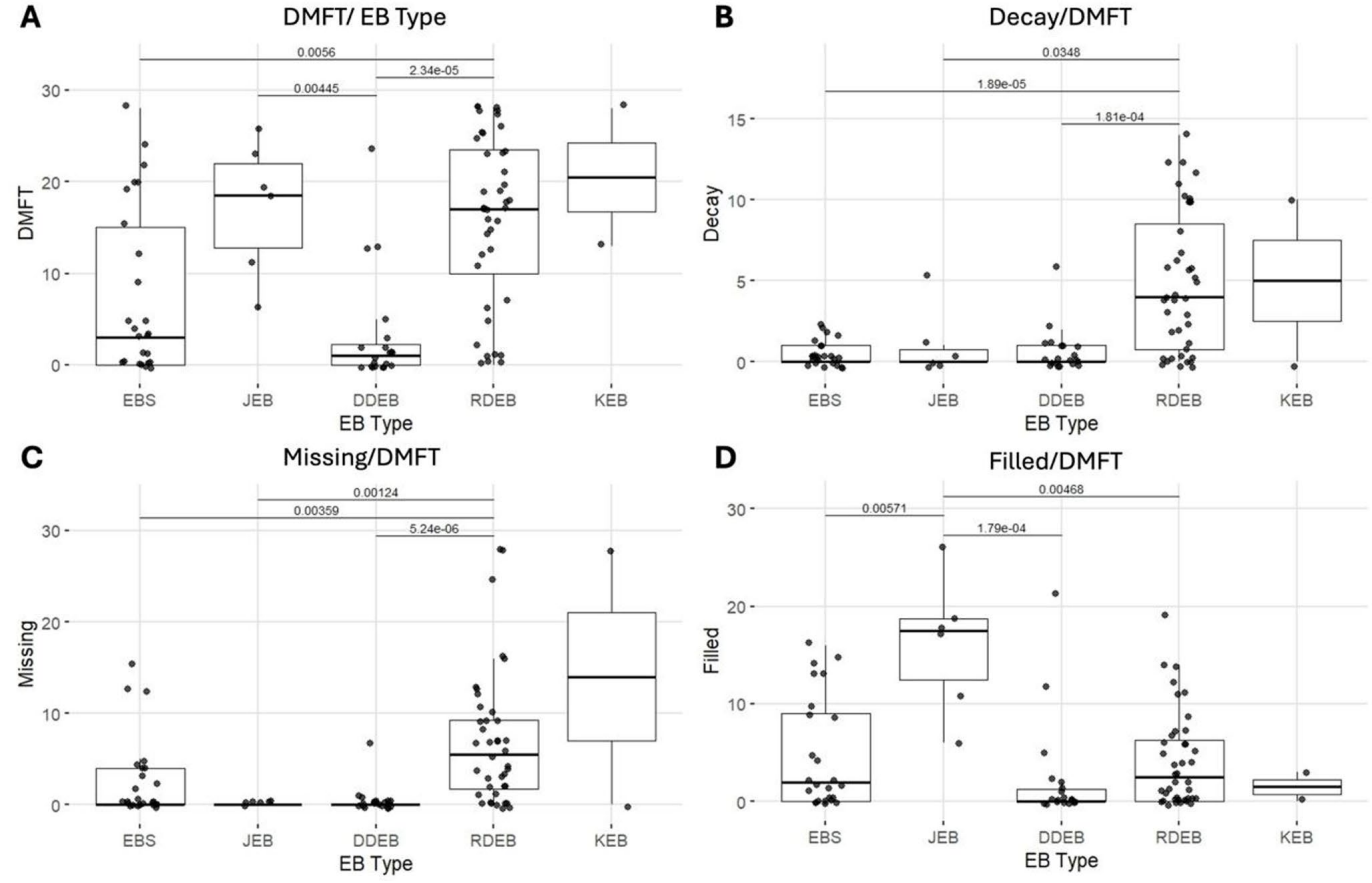
Boxplot showing distribution of DMFT index and each of its individual components per major type of EB. (**A**) Full DMFT index per EB type, (**B**) Caries per EB type, (**C**) Missing teeth per EB type and (**D**) Filled or restored teeth per EB type. Decay, missing, filled teeth index. Note: The data for KEB was not analysed statistically, due to the low number of participants

A total of 8 participants (7,9%) had deciduous dentition, 11 (10,9%) had mixed dentition, and 82 (81,1%) had permanent dentition. The total sample showed a DMFT of 11.2 ± 10.1, with JEB (17.2 ± 7.47) and RDEB (16 ± 9.33) having the highest scores (Fig. 2A) (p = 7.79e-06, Kruskal-Wallis/Dunn’s post-hoc). Decay (D) was higher in KEB (5 ± 7.07) and RDEB (4.85 ± 4.32) (p = 2.17e-06, Kruskal-Wallis/Dunn’s post-hoc) (Fig. 2B); Missing was also higher in KEB (14 ± 19.8) and RDEB (7.05 ± 7.38) (Fig. 2C) (p = 5.596e-07, Kruskal-Wallis/Dunn’s post-hoc), while Filling (F) was higher in JEB (16.2 ± 6.91), followed by RDEB (4.12 ± 4.86), with only about 25% of restorations when compared to JEB (Fig. 2D) (p = 5.027e-4, Kruskal-Wallis/Dunn’s post-hoc). All aspects showed significant values (p<0.05), as seen in Supplementary Table 4. KEB was excluded from the statistical analysis due to the low number of participants. For deciduous dentition, RDEB showed numerically higher decay values; however, these differences were not statistically significant (Supplementary Table 4).

### Orthodontics: Index of complexity, outcome and need (ICON)

The Index of Complexity, Outcome and Need (ICON) was assessed for 93 patients, excluding 9 with severely compromised aesthetic assessment due to missing teeth. After grading the 5 elements considered in this index, 64 patients (69.56%) had scores “lower than 43”, which is the threshold for determining treatment needs. Of the 28 patients with scores of 43 or higher, 16 (57.14%) presented RDEB, either the severe or intermediate subtype, 6 EBS (21,.42%), 4 DDEB (14.28%) and 2 JEB (7,14%), with no statistical difference between groups (*p* = 0.204; Kruskal-Wallis/Dunn’s post-hoc) (Supplementary Table 5).

### Dental treatment needs

**Fig. 3:**
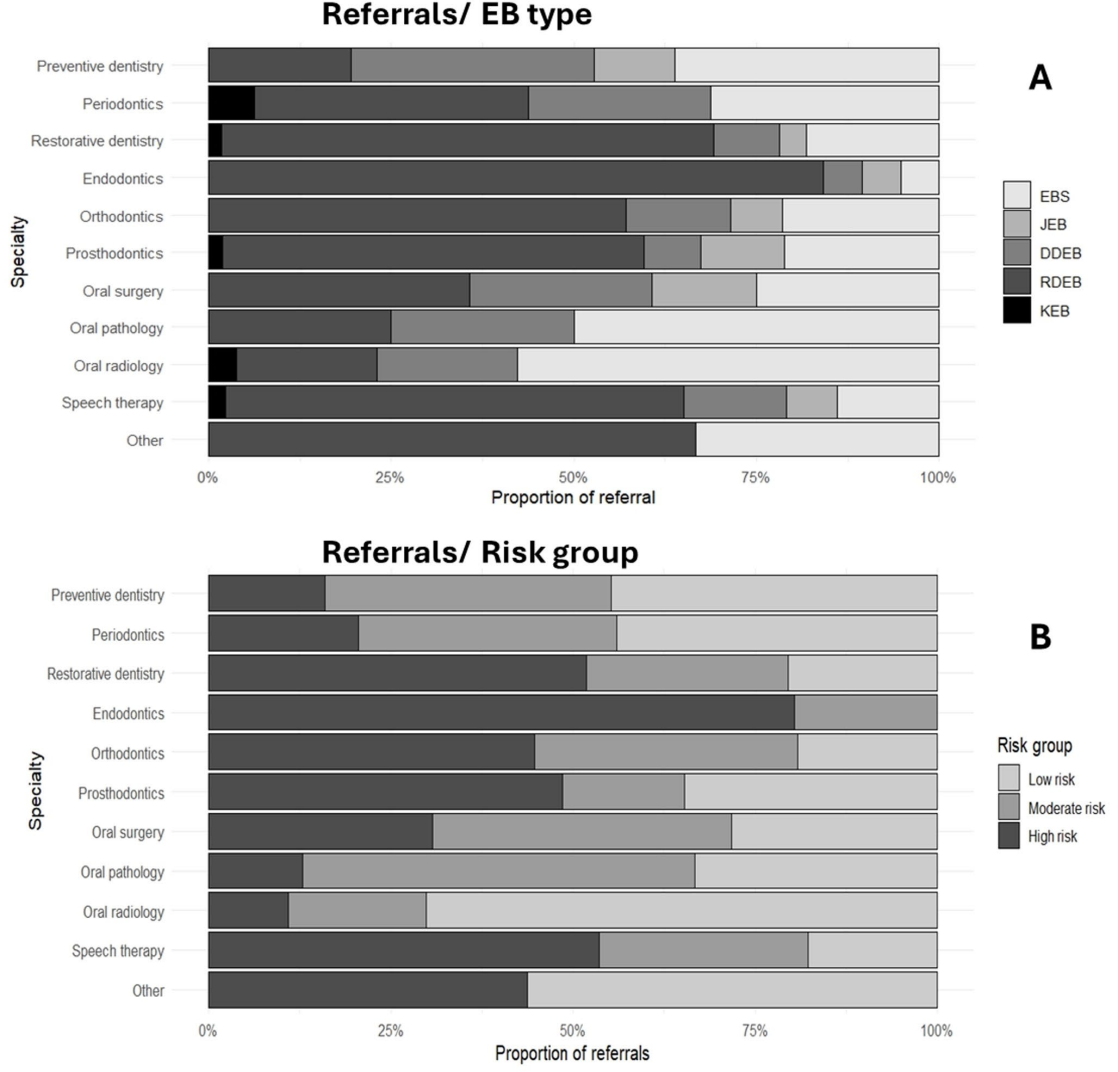
Proportion of referrals for each dental speciality. **A**) Referrals by EB type. **B**) Referrals by risk group according to Krämer et al[14]

Referrals for preventive dentistry, periodontics, restorative dentistry, endodontics, orthodontics, prosthodontics, surgery, oral pathology, oral radiology and speech and language therapy were recorded. In this cohort, referrals to paediatric dentistry and special care dentistry were not recorded, as it is understood that these professionals will be leading the long-term care of the patients. On average, patients from the cohort (*n* = 101) required a median of 3 different referrals, with the highest need being restorative dentistry (54.5%), prosthodontics (51.5%) and speech therapy (42.6%). The description for each EB type is shown in Table 2; Fig. 3A.

**Table 2:** Dental treatment needs in EB

PARTICIPANTS (*n*)	Median number of referrals per patient	Range	PREVENTIVE DENTISTRY *n*(%)	PERIODONTICS *n*(%)	RESTORATIVE DENTISTRY *n*(%)	ENDODONTICS *n*(%)	ORTHODONTICS *n*(%)	PROSTHODONTICS *n*(%)	SURGERY *n*(%)	ORAL PATHOLOGY *n*(%)	ORAL RADIOLOGY *n*(%)	SPEECH THERAPY *n*(%)	OTHER *n*(%)
by EB TYPE													
Total (*n* = 101)	3	1–5	36 (35.6)	16 (15.8)	55 (54.5)	19 (18.8)	28 (27.7)	52 (51.5)	28 (27.7)	4 (4.0)	26 (25.7)	43 (42.6)	3 (3.0)
EBS (*n* = 26)	3	1–5	13 (50)	5 (19.2)	10 (38.5)	1 (3.8)	6 (23.1)	11 (42.3)	7 (26.9)	2 (7.7)	15 (57.7)	6 (23.1)	1 (3.8)
JEB (*n* = 6)	4	3–4	4 (66.7)	0 (0)	2 (33.3)	1 (16.7)	2 (33.3)	6 (100)	4 (66.7)	0 (0)	0 (0)	3 (50)	0 (0)
DDEB (*n* = 20)	2	1–5	12 (60)	4 (20)	5 (25)	1 (5)	4 (20)	4 (20)	7 (35)	1 (5)	5 (25)	6 (30)	0 (0)
RDEB (*n* = 47)	3	1–5	7 (14.9)	6 (12.8)	37 (78.7)	16 (34)	16 (34)	30 (63.8)	10 (21.3)	1 (2.1)	5 (10.6)	27 (57.4)	2 (4.3)
KEB (*n* = 2)	2.5	1–4	0 (0)	1 (50)	1 (50)	0 (0)	0 (0)	1 (50)	0 (0)	0 (0)	1 (50)	1 (50)	0 (0)
by RISK OF ORAL DISEASE (According to Krämer et al. [14])													
High (*n* = 54)	3	1–5	11 (20.4)	6 (11.1)	39 (72.2)	17 (31.5)	18 (33.3)	36 (66.7)	14 (25.9)	1 (1.9)	6 (11.1)	31 (57.4)	2 (3.7)
Moderate (*n* = 26)	2	1–5	13 (50)	5 (19.2)	10 (38.5)	2 (7.7)	7 (26.9)	6 (23.1)	9 (34.6)	2 (7.7)	5 (19.2)	8 (30.8)	0 (0)
Low (*n* = 21)	3	1–5	12 (57.1)	5 (23.8)	6 (28.6)	0 (0)	3 (14.3)	10 (47.6)	5 (23.8)	1 (4.8)	15 (71.4)	4 (19)	1 (4.8)
p-value (Fisher’ exact test)	**/**	**/**	**5.02E-03**	6.11E-01	**2.36E-03**	**2.81E-03**	2.12E-01	**2.81E-03**	7.65E-01	5.63E-01	**1.29E-05**	**7.52E-03**	7.77E-01

***Abbreviations***: *EB* epidermolysis bullosa, *EBS *EB Simplex, *JEB* Junctional EB, *DDEB* Dominant Dystrophic EB, *RDE*B Recessive Dystrophic EB, *KEB* Kindler EB

When analysed by risk of oral disease, it can be observed that the groups “low” and “moderate” risk of oral disease have a higher need for preventive dentistry and oral radiology, in contrast with the group “high risk of oral disease”, which has a higher need for restorative dentistry and prosthodontics. Significant differences (p<0.05, Fisher’s exact test) were found in preventive dentistry, restorative dentistry, endodontics, prosthodontics and speech therapy (Table 2; Fig. 3B).

Each dental speciality was also analysed for age and gender through a binary logistic regression, estimating OR (95% CI) and p-values with Bonferroni correction. For age, a higher age was positively associated with higher needs for periodontics (OR 1.10, p-adj: 0.000315) and prosthodontics (OR: 1.13; p-adj: 0.0000674). For gender, the only significant value was for speech therapy, where men showed a higher association, with an OR of 3.83, p-adj = 0.0173. After applying Bonferroni correction, the initially significant p-values between surgery and men, as well as between age (younger) and preventive dentistry, orthodontics, surgery and speech therapy, were no longer significant (Table 3)

**Table 3:** Logistic regression for age and gender analysis

Dental Speciality	AGE			GENDER		
	OR (95% CI)	p-value	p-adj	OR (95% CI)	*p*-value	p-adj
Preventive dentistry	0.961	0.0181	0.2	0.617	0.253	1
Periodontics	1.1	0.0000287	0.000315	0.921	0.881	1
Restorative dentistry	0.992	0.542	1	1.47	0.336	1
Endodontics	1.01	0.408	1	1.04	0.936	1
Orthodontics	0.965	0.0688	0.757	0.556	0.208	1
Prosthodontics	1.13	0.00000613	0.0000674	0.599	0.203	1
Surgery	0.964	0.0453	0.499	2.73	0.0294	0.323
Oral Pathology	0.936	0.244	1	1.16	0.887	1
Oral Radiology	1.01	0.34	1	0.642	0.34	1
Speech Therapy	0.957	0.00727	0.08	3.83	0.00158	0.0173
Other	1.02	0.632	1	0.565	0.646	1

Age: OR higher for older age. Gender: OR higher for males. p-adj: *p*-values adjusted using Bonferroni correction for 11 dental specialities

## Discussion

**Fig. 4:**
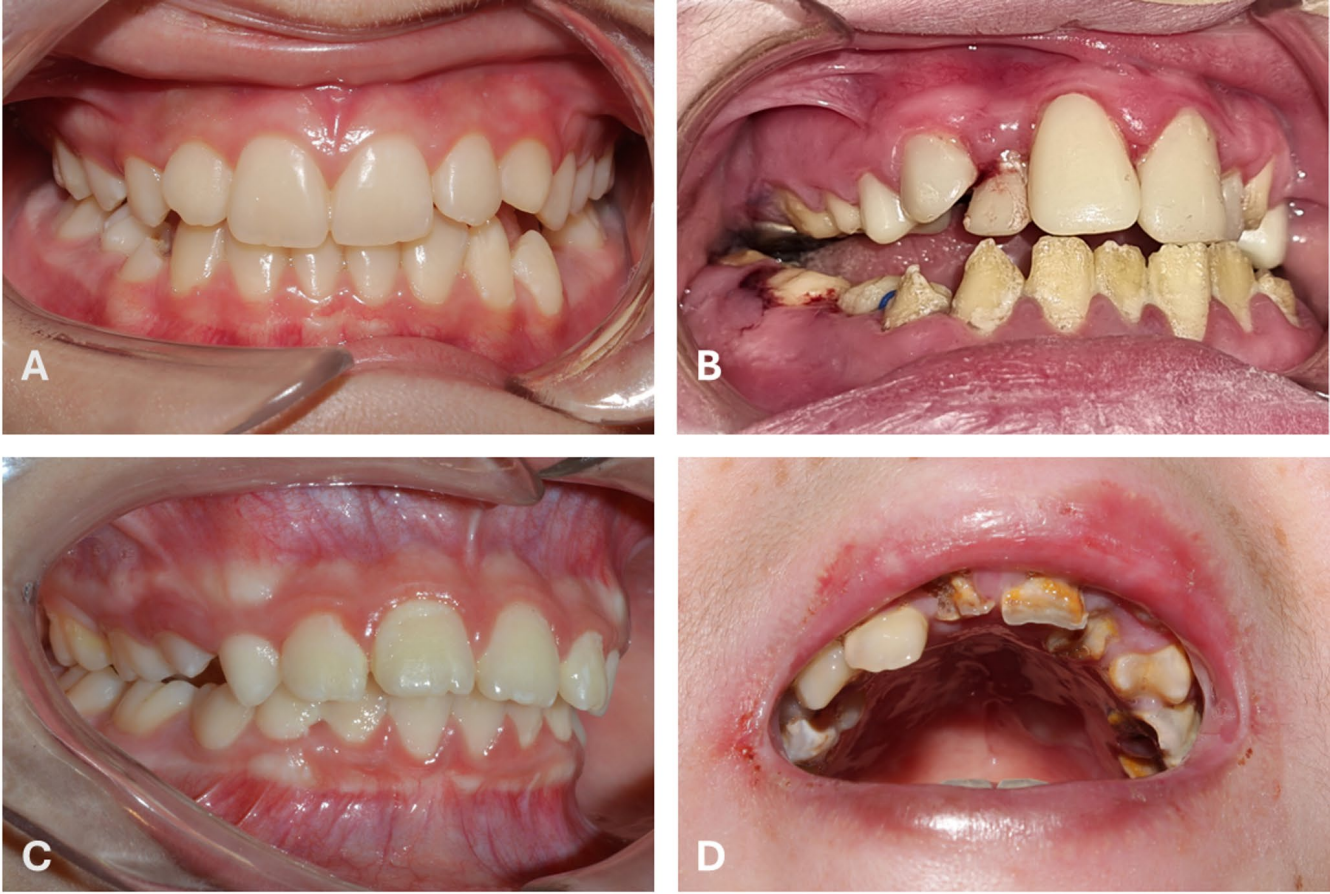
Treatment needs in different EB types. **A**) A 11-year-old female patient with EBS due to a heterozygous genetic variant in KRT5 (c.1283C>T). Requires preventive dentistry and orthodontics. **B**) A 16-year-old male patient with JEB due to a homozygous genetic variant in LAMB3 (C.3228+1G>A). Requires periodontal treatment and surgery due to gingival hyperplasia, orthodontic and oral full mouth oral rehabilitation due to amelogenesis imperfecta. **C**) A 13-year-old female patient with DDEB due to a heterozygous variant in COL7A1 (C.5318G>T ). Requires preventive dentistry and orthodontics. **D**) A 14-year-old male patient with RDEB due to a compound heterozygous genetic variant in COL7A1 (C.7708delG y C.2005C>T). The patient did not attend regularly to dental check-ups and presented poor oral hygiene with gingivitis and multiple severe cavities. Treatment needs included preventive and restorative dentistry, endodontics, oral rehabilitation, and dental radiographs

This article characterised the oral status and dental treatment needs of a heterogeneous cohort of patients with different types of Epidermolysis Bullosa (EB) registered at a national reference centre. The cohort included patients with all major types of EB in a wide age range (2 to 74 years, average 22.2 years). This heterogeneity is clinically relevant, as EB is a lifelong condition with a wide clinical spectrum, and severe cases present cumulative health conditions with consequences across the lifespan, such as oral strictures, amelogenesis imperfecta, and pseudosyndactyly (Fig. 4). Caries experience is also cumulative and can be influenced by factors including age, disease severity and historical dental treatment. Therefore, the findings in this study should be interpreted as a comprehensive overview of oral health challenges in EB rather than an age-specific approach.

Oral hygiene, a fundamental pillar of oral health, was worse in individuals with RDEB (average DI-S values 2.34 ± 0.7), with significant differences (*p*<0.05) when compared to DDEB (1.53 ± 0.8). This can be explained by various factors, such as the severity of mucosal fragility, microstomia, pain and pseudosyndactyly [6, 34]. Heavy plaque and calculus accumulation in RDEB have been previously reported, mostly in the lingual and buccal surface of mandibular posterior teeth [6, 35]. Some suggestions on how to facilitate brushing include small headed toothbrushes or specific toothbrushes such as Collis Curve^®^ toothbrush, and Dr Bardman’s Superbrush^®^ [6, 36]. Additionally, from a research perspective, the difference between patients with RDEB and DDEB is important. Although both subtypes are caused by pathogenic genetic variants in the same gene (*COL7A1)*, the genotype-phenotype relationship with distinct inheritance patterns results in different phenotypic expressions. Harris et al. had previously reported higher values for plaque index (O’Leary) in a group of dystrophic EB (DEB) participants (*n* = 23, 18 RDEB and 5 DDEB) when compared to a control group [37]. But did not differentiate between RDEB and DDEB. Our results suggest that it would not be appropriate to consider a DEB as a whole group. 

Oral hygiene, a fundamental pillar of oral health, was worse in individuals with RDEB (average DI-S values 2.34 ± 0.7), with significant differences (*p*<0.05) when compared to DDEB (1.53 ± 0.8). This can be explained by various factors, such as the severity of mucosal fragility, microstomia, pain and pseudosyndactyly [6, 34]. Heavy plaque and calculus accumulation in RDEB have been previously reported, mostly in the lingual and buccal surface of mandibular posterior teeth [6, 35]. Some suggestions on how to facilitate brushing include small headed toothbrushes or specific toothbrushes such as Collis Curve^®^ toothbrush, and Dr Bardman’s Superbrush^®^ [6, 36]. Additionally, from a research perspective, the difference between patients with RDEB and DDEB is important. Although both subtypes are caused by pathogenic genetic variants in the same gene (*COL7A1)*, the genotype-phenotype relationship with distinct inheritance patterns results in different phenotypic expressions. Harris et al. had previously reported higher values for plaque index (O’Leary) in a group of dystrophic EB (DEB) participants (*n* = 23, 18 RDEB and 5 DDEB) when compared to a control group [37]. But did not differentiate between RDEB and DDEB. Our results suggest that it would not be appropriate to consider a DEB as a whole group.

The poor values in oral hygiene are reflected in the BPE assessment, with a high prevalence of gingivitis and only 7% of the participants with healthy values (BPE 0). Gingivitis (BPE 1 or 2) has been extensively reported in RDEB [6], while gingival hyperplasia has been reported in JEB [6, 12]. Periodontal disease (BPE 3 or 4), was diagnosed in 14 of 99 (14.1%) participants and was evenly distributed across EB types. The need for periodontal treatment was significantly associated with the age of the participants (OR 1.01; p-adj: <0.001), an association similar to the non-EB population [38]. This apparent discrepancy may be explained by the chronic nature of periodontal disease, the younger age of many patients with severe EB and the reduced number of teeth [6]. Patients with KEB have an increased risk of early-onset periodontal disease [12]. Our cohort only included two patients with this rare subtype of EB, one of them being completely edentulous at the age of 39, which could have been a result of early-onset periodontal disease, but no clinical records were available to confirm this. A limitation of the methodology used in this study to assess periodontal features is the lack of periodontal characterisation. This is relevant, as specific EB types are associated with other periodontal manifestations, such as JEB, which is linked to gingival enlargement [6, 12, 39]. Future studies characterising the gingival tissues should screen for gingival overgrowth/enlargement as well.

In contrast to periodontal findings, caries experience and DMFT values showed a stronger association with severe EB subtypes. The DMFT index should be interpreted carefully in this research, as restored teeth due to amelogenesis imperfecta can increase the “filled” component of the index and be mistakenly interpreted as caries. Indeed, the highest DMFT scores in this cohort were observed in JEB (17.2 ± 7.47). Participants with JEB presented SAI due to LAMB3 variants. Most participants had a history of hypersensitivity, attrition, crown resorption and therefore required major oral rehabilitation [16, 40], which explains the high number of fillings in this group (16.2 ± 6.91, Fig. 2D). The second-highest DMFT score was observed on patients with RDEB (16 ± 9.33). Participants with RDEB showed significantly higher values for Decay (4.85 ± 4.32) and Missing (7.05 ± 7.38), Fig. 2B and C, both in deciduous and permanent dentition, which is in accordance with previously published data [6, 34, 37, 39, 41]. Patients with RDEB present several characteristics that increase the risk of caries, such as oral strictures, a soft diet and pseudosyndactyly, which limit oral hygiene maintenance [6]. The DMFT index for KEB was not statistically compared to the other types of EB, as only 2 patients were examined. However, its results show a high number of missing teeth. This also has to be interpreted carefully, as this might not reflect teeth lost due to caries, but is associated with early-onset periodontal diseases (Fig. 2C).

A novel contribution from this research is the establishment of the orthodontic treatment needs for the EB population, measured with the ICON Index. This index has been applied in several studies in the healthy population [42, 43] and also for special care patients such as individuals with autism [44]. In this cohort, approximately one quarter of patients presented orthodontic treatment needs. This is slightly lower than the report by Liepa et al. with the same instrument, who reported an overall prevalence of orthodontic treatment needs of 35,3% in a cohort of healthy children in Latvia using the ICON. This could be explained by the presence of an orthodontist in our EB team, showing only the unmet treatment needs on this topic [42]. The ICON, while helpful, has certain limitations, including the high weight assigned to the aesthetic component [45], and it only considers upper arch crowding, excluding lower arch crowding. Moreover, since the index has not been validated for this particular condition, it is challenging to determine the treatment difficulty accurately. This categorisation does not account for EB-specific factors such as microstomia, vestibule obliteration, ankyloglossia, amelogenesis imperfecta, and skin fragility, meaning the treatment difficulty is likely underestimated for severe EB subtypes.

Evidence regarding dental treatment needs in EB is scarce, and, to our knowledge, only one study has explored the needs for dental treatment in participants with EB: a scoping review focused on children, reported by Smith et al. in 2024. This research stated that most children require preventive treatment, followed by restorative treatment and a combination of both, highlighting the importance of early access and proper referral pathways [26]. Our research adds to this topic the adults’ needs, with significant needs for Periodontics and Prosthodontics.

Additionally, all patients in our cohort had granted access to dental care in the national reference centre and were under the regular care of a paediatric dentist, special care dentist and/or an orthodontist. Despite access to dental care, participants showed a high burden of oral disease. This could be explained by different barriers related to EB, including physical (mucosal fragility, pain, microstomia, etc.) and psychosocial (long distance from the reference centre, personal motivations, etc.) barriers [14].

Additionally, the results of this research highlight the complexity of dental care in EB, especially for those at high risk, where at least 3 to 4 different dental areas are required, complementing the referral pathways for people living with EB published by Krämer et al. [14]. Many recommendations and guidelines highlight the importance of multidisciplinary and early interventions in EB [6, 14], but information on the specific treatment needs is scarce. In many countries, dental care for EB patients is mostly provided by a paediatric dentist or a special care dentist, with limited access to other specialities [14]. Healthcare providers and health insurance should consider including specialities such as prosthodontics in the basic coverage of those with a high risk of oral disease, as well as access to consultants in other dental specialities. This also should include non-dental areas, such as speech therapy, especially for those at high risk, and access to a specialised team is fundamental for proper oral care [14, 26]. Overall, our findings highlight that oral disease burden in EB varies according to the major type and cannot solely be explained by access to care. 

## Limitations

This article presents some limitations that must be addressed. First, due to the reduced prevalence of KEB, it is difficult to include this group in the statistical analysis. Our cohort only included two patients with KEB; thus, their data were presented descriptively but not included in the statistical analysis. Collaborative approaches between centres must be considered to increase this sample. Missing data also reduced the effective sample size for some outcomes, which may have decreased the statistical power to detect significant associations (the highest missing data was 8 out of 101 in the ICON analysis. Complete information can be observed on Flowchart suppl. Figure 1). Due to the limited number of potential participants, imputation was not appropriate and complete-case analysis was used; therefore, non-significant findings should be interpreted with caution. 

A second limitation is that only participants who attended at least one check-up during the recruitment period could be assessed. Therefore, a potential selection bias would be that patients in the register not interested in having a free dental examination were not actively contacted or invited to participate. Additionally, blinding of the clinical examiners and settings was not feasible, due to the nature of the condition. Although examiner calibration and standardised criteria were applied, observer bias cannot be completely excluded for outcomes based on clinical judgement such as referral needs.

A third point that needs to be highlighted when analysing this data is that our cohort has access to free dental care at a national reference centre; therefore, most patients with a severe risk of oral disease attend regular dental care, contrary to those with moderate or low risk. The fact that patients with a high risk of oral disease are under a close follow-up programme may play a role in these results. This can lead to bias in aspects such as the need for oral radiology. For example, none of the six patients with JEB needed a dental radiograph, because all are in close follow-up and already had panoramic radiographs taken within the last year. Similarly, the need for restorative dentistry, only two out of six needed restorative treatment (Table 2), because all others had their entire dentition already crowned. In a similar perspective, only 7% of patients with RDEB needed preventive dentistry. This is because all others had all the preventive strategies well covered. It has to be stated: all patients with RDEB do require a close preventive program, all patients with JEB will need radiographs and restorative dentistry, although in our cohort, it is not highlighted as an unmet need. In addition, the need for orthodontic treatment in this cohort was extremely low (20% to 34%). This is also associated with the fact that the orthodontist is part of the clinic’s permanent team and follows the craniofacial development of all patients who regularly attend the clinic. The low orthodontic treatment need in our cohort probably reflects the high orthodontic treatment provision, not a low need within patients with EB. This data has to be analysed with caution and may not be extrapolated to other cohorts where orthodontists are not part of the regular dental team. Finally, as a cross-sectional study, this research presents limitations inherent to the study design, including the inability to establish causality and the potential for selection bias.

## Conclusions

Oral health status among people living with EB differed across major types. Patients with recessive dystrophic EB were associated with poorer oral hygiene, a higher caries experience and more missing teeth, whereas those with junctional EB were associated with a higher number of restored teeth. On the opposite, those with simplex EB and dominant dystrophic EB were associated with more favourable oral health indicator. 

The greatest referral needs were for restorative dentistry, prosthodontics and speech therapy, with higher referral needs observed among EB subtypes with high risk of oral disease (recessive dystrophic EB, junctional EB and Kindler EB). Older age was associated with periodontics and prosthodontics referral needs, while male participants were associated with a higher need for speech therapy. 

## Supplementary material

Below is the link to the electronic supplementary material.

Supplementary File 1 (DOCX 234 KB)

## Data Availability

The data underlying this article will be shared on reasonable request to the corresponding author.
