# Supplementary material for: Oral status and dental treatment needs in patients with Epidermolysis Bullosa - a cross-sectional study
Source: Clin Oral Investig. 2026 Apr 10;30(5):169. doi: 10.1007/s00784-026-06779-x (PMC13065552; doi:10.1007/s00784-026-06779-x)
Supplement: Supplementary file 1 — (DOCX 234 KB) [file 784_2026_6779_MOESM1_ESM.docx]

**SUPPLEMENTARY DATA**

**Supplementary Table 1: Treatment needs parameters.**

| Description | Parameters |
| --- | --- |
| Preventive dentistry | Routine dental check-up, educational counselling, pits and fissures sealants, fluoride, and professional basic cleaning including plaque removal in periodontal pockets <3.5 mm (BPE:2). |
| Periodontics | Periodontal pockets >3.5 mm (BPE: 3 and 4), furcation involvement, periodontal defects or conditions that require periodontal surgery, or patients with a medical condition not EB-related that affects the periodontal tissue. Those participants with BPE 3 or 4 were assessed later with the current periodontal classification. |
| Restorative | Caries or lesions (for example, hypoplasia) that require simple or complex filling without major oral rehabilitation (Decay from DMFT Index), including teeth that require endodontic treatment and later restorative treatment. |
| Endodontics | Teeth with specific endodontic referral due to deep caries, retreatment or crown restoration. |
| Orthodontics | ICON Index for establishing orthodontic treatment needs [33]. |
| Oral rehabilitation/ Prosthodontics | Includes prosthodontics and implantology: rehabilitation of missing teeth (Missing from the DFMT Index) and teeth that require endodontic treatment with subsequent major treatment, such as crowns or bridges. |
| Surgery | Teeth extractions, orthognathic surgery, and management of impacted or included teeth. |
| Oral Pathology | Presence of non-EB-related lesions that may require biopsy. |
| Radiology | Need for an up-to-date radiograph based on individual clinical findings.  Note: At the national reference centre, most EB patients with a high risk of oral diseases have one orthopantomography (OPG) taken on a yearly basis and attend follow up appointments every 3 months. Therefore, those patients who already had OPG taken in the last years were not registered as needing new imaging studies. |
| Speech Therapy | Functional problems, including self-reported bruxism, deglutition limitations due to strictures such as ankyloglossia or oesophageal problems, including a history of oesophageal dilations. |
| Other | Any other professional who might be required to treat a specific condition, including chronic pain due to bruxism or temporo-mandibular complications. |

**Abbreviations:** BPE: Basic Periodontal Examination; DMFT: Decay-Missing-Filled Teeth Index. ICON: orthodontic index of complexity, outcome and need.

**Supplementary Table 2: Debris Index - Simplified (DI-S).**

| Participants (n) | | Debris Index - Simplified [29] | | | | | | | |
| --- | --- | --- | --- | --- | --- | --- | --- | --- | --- |
|  |  | M | DI-S  (Av ± SD) | Median | Min | Max | Good  [ n (%)] | Regular [ n (%)] | Bad  [ n (%)] |
| Total (n=101) | | 3 | 2.04 ± 0.83 | 2.08 | 0.00 | 3.00 | 3 (3.0) | 32 (32.7) | 63 (64.3) |
|  | EBS (n=26) | 0 | 1.98 ± 0.81 | 1.83 | 0.17 | 3.00 | 1 (3.8) | 10 (38.5) | 15 (57.7) |
|  | JEB (n=6) | 0 | 1.86 ± 0.96 | 1.58 | 1.00 | 3.00 | 0 (0) | 3 (50.0) | 3 (50.0) |
|  | DDEB (n=20) | 0 | 1.53 ± 0.80 | 1.33 | 0.00 | 3.00 | 2 (10.0) | 10 (50.0) | 8 (40.0) |
|  | RDEB (n=47) | 2 | 2.34 ± 0.73 | 2.67 | 0.83 | 3.00 | 0 (0) | 8 (17.8) | 37 (82.2) |
|  | KEB (n=2) | 1 | 1.00 | 1.00 | 1.00 | 1.00 | NA | 1 (100) | NA |

**Abbreviations:** EB, epidermolysis bullosa; EBS: EB Simplex; JEB: Junctional EB, DDEB: Dominant Dystrophic EB; RDEB: Recessive Dystrophic EB; KEB: Kindler EB. M: Missing; DI-S: Debris Index- Simplified, Av: Average, SD: Standard deviation; Good: (<0.7); Regular: 0.7-1.8, Bad: >1.8.

**Supplementary Table 3: Periodontal assessment.**

| Participants (n) | | Periodontal assessment | | | | | | | |
| --- | --- | --- | --- | --- | --- | --- | --- | --- | --- |
|  |  | Basic Periodontal Examination (BPE) [30] | | | | | | Periodontal stage and grade [31] | |
|  |  | M | 0  [n (%)] | 1  [n (%)] | 2  [n (%)] | 3  [n (%)] | 4  [n (%)] | V | Stage and Grade |
| Total (n=101) | | 2 | 7 (7.0) | 20 (20.2) | 57 (57.6) | 12 (12.1) | 3 (3.0) | 15 | / |
|  | EBS (n=26) | 0 | 2 (7.7) | 2 (7.7) | 17 (65.4) | 3 (11.5) | 2 (7.7) | 5 | 1B, 2B, 2B, 3B, 4C |
|  | JEB (n=6) | 0 | 1 (16.7) | 2 (33.3) | 3 (50.0) | 0 (0) | 0 (0) | 0 | / |
|  | DDEB (n=20) | 0 | 3 (15.0) | 6 (30.0) | 7 (35.0) | 3 (15.0) | 1 (5.0) | 4 | GH, 2B, 2B, 4C |
|  | RDEB (n=47) | 1 | 1 (2.1) | 10 (21.7) | 30 (65.2) | 6 (13.0) | 0 (0) | 6 | 1A, 1A, 1A, 2B, 2B, 2C |
|  | KEB (n=2) | 1 | 0 (0) | 0 (0) | 1 (100) | 0 (0) | 0 (0) | 0 | / |

**Abbreviations:** EB, epidermolysis bullosa; EBS: EB Simplex; JEB: Junctional EB, DDEB: Dominant Dystrophic EB; RDEB: Recessive Dystrophic EB; KEB: Kindler EB. M: Missing; V: Valid.GH: Gingival hyperplasia.

**Supplementary Table 4: Decayed-Missing-Filled Teeth Index (DMFT)**

|  | | Decayed - Missing - Filled Teeth index (DMFT/deft) | | | | | | | |
| --- | --- | --- | --- | --- | --- | --- | --- | --- | --- |
|  |  | Total (n=101) | EBS (n=26) | JEB (n=6) | DDEB (n=20) | RDEB (n=47) | KEB (n=2) | KW (p-value) | Dunn - p (adj) <.05 |
| V | | 93 | 25 | 6 | 20 | 40 | 2 | 91 |  |
| DMFT (Av ± SD) | | 11.2 ± 10.1 | 7.76 ± 9.23 | 17.2 ± 7.47 | 3.3 ± 6.25 | 16 ± 9.33 | 20.50 ± 10.61 | **7.79e-06** | DDEB - JEB= 4.45e-3 DDEB - RDEB = 2.34e-5 EBS - RDEB =5.60e-03 |
|  | *Median (R)* | 11 (1–20) | 3 (0–15) | 18.5 (12.75–22) | 1 (0–2.25) | 17 (10–23.5) | 20.5 (16.75–24.25) |  |  |
|  | *Min - Max* | 0-28 | 0-28 | 6-26 | 0-24 | 0-28 | 13-28 |  |  |
| D (Av ± SD) | | 2.52 ± 3.71 | 0.44 ± 0.77 | 1 ± 2 | 0.65 ± 1.39 | 4.85 ± 4.32 | 5 ± 7.07 | **2.17e-06** | DDEB - RDEB = 1.81e-4 EBS - RDEB = 1.89e-5 JEB-RDEB= 3.48e-2 |
| M (Av ± SD) | | 4.13 ±6.58 | 2.6 ± 3.8 | 0 | 0.45 ± 1.57 | 7.05 ± 7.38 | 14 ± 19.8 | **5.596e-07** | DDEB - RDEB=5.24e-6 EBS - RDEB= 3.59e-3 JEB - RDEB = 1.24e-3 |
| F (Av ± SD) | | 4.59 ± 6.09 | 4.72 ± 5.7 | 16.2 ± 6.91 | 2.2 ± 5.25 | 4.12 ± 4.86 | 1.5 ± 2.12 | **5.027e-4** | DDEB - JEB= 1.79e-4 EBS - JEB =5.71e-3  JEB - RDEB= 4.68e-3 |
| V | | 19 | 4 | 1 | 3 | 11 | 0 | 19 |  |
| deft (Av ± SD) | | 4 ± 4.56 | 5.75 ± 6.08 | 5 ± NA | 0 ± 0 | 4.36 ± 4.48 | / | 0.2092 | / |
|  | *Median (R)* | 2 (0–8) | 6 (0.75–11) | 5 (5–5) | 0 (0–0) | 3 (0.5–8) | / |  |  |
|  | *Min - Max* | 0-12 | 0-11 | 5-5 | 0-0 | 0-12 | / |  |  |
| d (Av ± SD) | | 2.68 ± 3.77 | 5.75 ± 6.65 | 0 ± NA | 0 ± 0 | 2.55 ± 2.38 | / | 0.21 | / |
| e (Av ± SD) | | 0.05 ± 0.23 | 0 | 0 | 0 | 0.09 ± 0.3 | / | 0.8668 | / |
| f (Av ± SD) | | 1.32 ± 2.06 | 0.25 ± 0.5 | 5 | 0 | 1.73 ± 2.24 | / | 0.1357 | / |

**Abbreviations:** EB, epidermolysis bullosa; EBS: EB Simplex; JEB: Junctional EB, DDEB: Dominant Dystrophic EB; RDEB: Recessive Dystrophic EB; KEB: Kindler EB. V: Valid; DMFT: Decayed-Missing-Filled Teeth Index for permanent dentition, deft: decayed-extracted-filled teeth index in primary dentition. D/d: Decay, M: Missing, e: Extracted, F/f: Filled, Av: Average, SD: Standard deviation; Med: Median, R: Range, KW: Kruskal-Wallis test.

**Supplementary Table 5: Orthodontics: ICON Index [33].**

| EB CLASSIFICATION | | | | | ICON - TREATMENT COMPLEXITY INDEX | | | | | TREATMENT NEED | |
| --- | --- | --- | --- | --- | --- | --- | --- | --- | --- | --- | --- |
| EB TYPE | EB SUBTYPE | N | V | M | Easy [N (%)] | Mild [N (%)] | Moderate [N (%)] | Difficult [N (%)] | Very difficult [N (%)] | No need [N (%)] | Need [N (%)] |
| EBS | LOCALISED | 9 | 8 | 1 | 3 (37.5) | 4 (50.0) | 0 (0) | 1 (12.5) | 0 (0) | 6 (75.0) | 2 (25.0) |
|  | INTERMEDIATE | 12 | 10 | 2 | 8 (80.0) | 1 (10.0) | 0 (0) | 0 (0) | 1 (10.0) | 9 (90.0) | 1 (10.0) |
|  | INTERMEDIATE WITH MD | 5 | 5 | 0 | 2 (40.0) | 0 (0) | 2 (40.0) | 1 (20.0) | 0 (0) | 2 (40.0) | 3 (60.0) |
|  | TOTAL EBS | 26 | 23 | 3 | 13 (56.5) | 5 (21.7) | 2 (8.7) | 2 (8.7) | 1 (4.3) | 17 (73.9) | 6 (26.1) |
| JEB | INTERMEDIATE | 3 | 3 | 0 | 1 (33.3) | 1 (33.3) | 1 (33.3) | 0 (0) | 0 (0) | 1 (33.3) | 2 (66.6) |
|  | SEVERE | 3 | 3 | 0 | 3 (100) | 0 (0) | 0 (0) | 0 (0) | 0 (0) | 3 (100) | 0 (0) |
|  | TOTAL JEB | 6 | 6 | 0 | 4 (66.6) | 1 (16.6) | 1 (16.6) | 0 (0) | 0 (0) | 4 (66.6) | 2 (33.3) |
| DDEB | LOCALISED | 14 | 14 | 0 | 9 (64.2) | 1 (7.1) | 1 (7.1) | 3 (21.4) | 0 (0) | 10 (71.4) | 4 (28.5) |
|  | INTERMEDIATE | 2 | 2 | 0 | 1 (50.0) | 1 (50.0) | 0 (0) | 0 (0) | 0 (0) | 2 (100) | 0 (0) |
|  | PRURIGINOSA | 4 | 4 | 0 | 4 (100) | 0 (0) | 0 (0) | 0 (0) | 0 (0) | 4 (100) | 0 (0) |
|  | TOTAL DDEB | 20 | 20 | 0 | 14 (70.0) | 2 (10.0) | 1 (5.0) | 3 (15.0) | 0 (0) | 16 (80.0) | 4 (20.0) |
| RDEB | LOCALISED | 1 | 1 | 0 | 1 (100) | 0 (0) | 0 (0) | 0 (0) | 0 (0) | 1 (100) | 0 (0) |
|  | INTERMEDIATE | 17 | 16 | 1 | 7 (43.7) | 4 (25.0) | 4 (25.0) | 1 (6.2) | 0 (0) | 10 (62.5) | 6 (37.5) |
|  | SEVERE | 27 | 25 | 2 | 6 (24.0) | 10 (40.0) | 5 (20.0) | 4 (16.0) | 0 (0) | 15 (60.0) | 10 (40.0) |
|  | INVERSA | 2 | 1 | 1 | 1 (100) | 0 (0) | 0 (0) | 0 (0) | 0 (0) | 1 (100) | 0 (0) |
|  | TOTAL RDEB | 47 | 43 | 4 | 15 (34.8) | 14 (32.5) | 9 (20.9) | 5 (11.6) | 0 (0) | 27 (62.7) | 16 (37.2) |
| KEB | KINDLER | 2 | 1 | 1 | 0 (0) | 1 (100) | 0 (0) | 0 (0) | 0 (0) | 1 (100) | 0 (0) |
|  | TOTAL | 101 | 93 | 8 | 46 (49.5) | 23 (24.7) | 13 (14.0) | 10 (10.8) | 1 (1.0) | 65 (69.9) | 28 (30.1) |

**Abbreviations:** EB, epidermolysis bullosa; EBS: EB Simplex; JEB: Junctional EB, DDEB: Dominant Dystrophic EB; RDEB: Recessive Dystrophic EB; KEB: Kindler EB; MD: Intermediate with muscular dystrophy, V: Valid; M: Missing.

**Supplementary Figure 1: Flowchart of participants and data.**


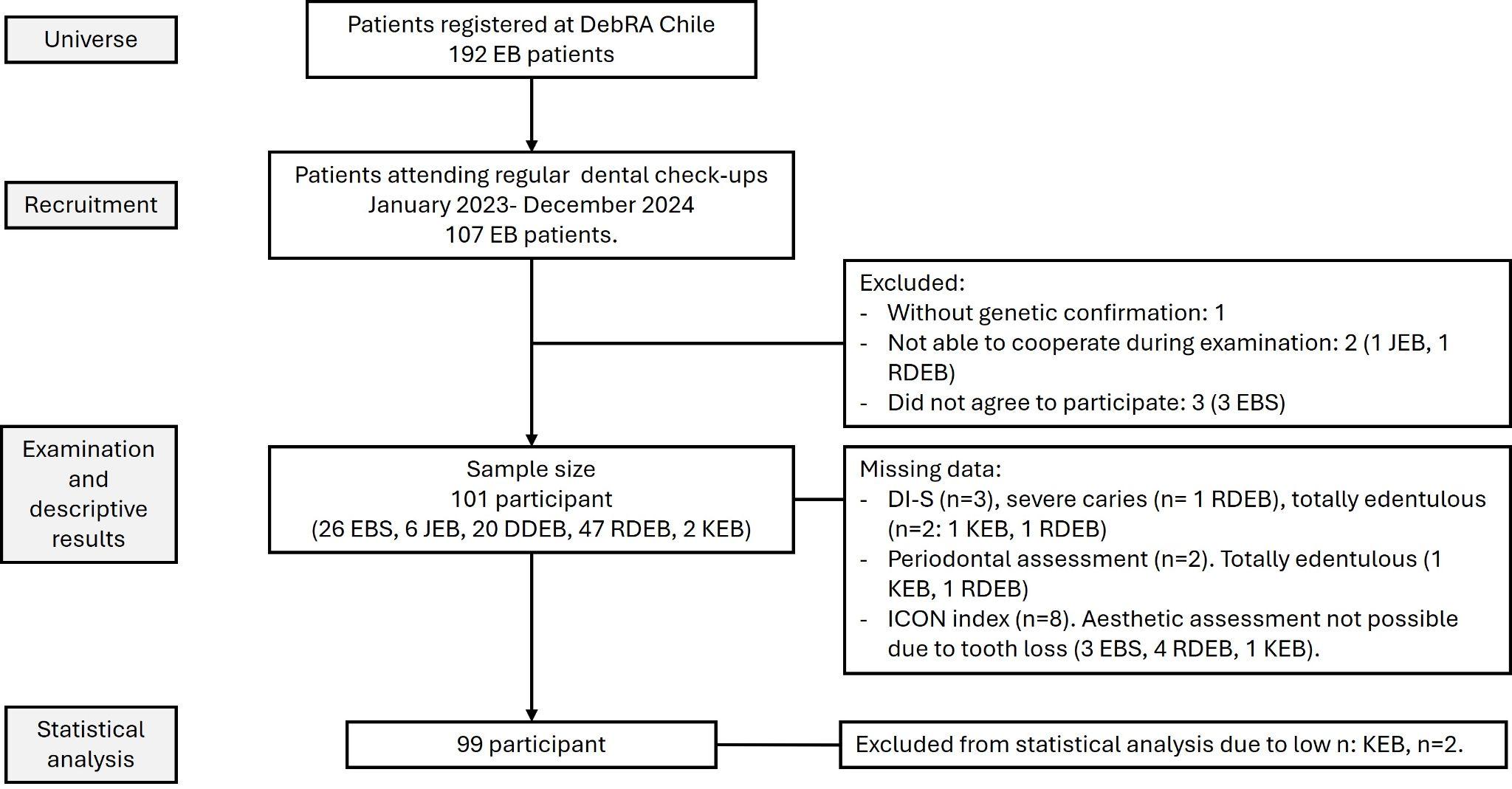


**Abbreviations:** EB, epidermolysis bullosa; EBS: EB Simplex; JEB: Junctional EB, DDEB: Dominant Dystrophic EB; RDEB: Recessive Dystrophic EB; KEB: Kindler EB.
